# Supplementary material for: LuminoCell: a versatile and affordable platform for real-time monitoring of luciferase-based reporters
Source: Life Sci Alliance. 2022 Apr 19;5(8):e202201421. doi: 10.26508/lsa.202201421 (PMC9018015; doi:10.26508/lsa.202201421)
Supplement: Supplementary file 1 [file LSA_LSA-2022-01421_TableS1.docx]

| Costs to build the LuminoCell | | |
| --- | --- | --- |
| **Quantity** | **Part** | **Price (USD)** |
| **6x** | **TSL237S-LF** | **24** |
| **1x** | **Arduino Nano Every** | **11** |
| **1x** | **Capacitor** | **0.1** |
| **1x** | **USB cable** | **4** |
| **Estimated total costs** | | **39.1** |

**Table S1:** Summary of costs to build the LuminoCell. Costs for 3D printed case are not included.
