# Supplementary material for: LuminoCell: a versatile and affordable platform for real-time monitoring of luciferase-based reporters
Source: Life Sci Alliance. 2022 Apr 19;5(8):e202201421. doi: 10.26508/lsa.202201421 (PMC9018015; doi:10.26508/lsa.202201421)
Supplement: Supplementary file 2 [file LSA_LSA-2022-01421-TableS2.docx]

| List of primary antibodies used for Western blot analysis | | |
| --- | --- | --- |
| Name | Catalogue Number | Manufacturer |
| Axin2 | 2151 | Cell Signaling Technology |
| β-Catenin | 05-665 | Merck |
| Phospho-β-Catenin (Ser33/37/Thr41) | 9564 | Cell Signaling Technology |
| β-Actin | 4970 | Cell Signaling Technology |
| Cleaved PARP | 9541 | Cell Signaling Technology |
| pERK1/2 | 9101 | Cell Signaling Technology |
| tERK1/2 | 4695 | Cell Signaling Technology |
| BMAL1 | ab93806 | Abcam |

**Table S2:** Summary of primary antibodies used in this study.
